# Colloidal Stability & Conformational Changes in β-Lactoglobulin: Unfolding to Self-Assembly

**DOI:** 10.3390/ijms160817719

**Published:** 2015-08-03

**Authors:** Steven Blake, Samiul Amin, Wei Qi, Madhabi Majumdar, E. Neil Lewis

**Affiliations:** Malvern Instruments, 7221 Lee Deforest Drive, Suite 300, Columbia, MD 21046, USA; E-Mails: steve.blake@malvern.com (S.B.); wei.qi@malvern.com (W.Q.); madhabi.majumdar@malvern.com (M.M.); neil.lewis@malvern.com (E.N.L.)

**Keywords:** dynamic light scattering, differential scanning calorimetry, Raman spectroscopy, self-assembly, protein aggregation, protein unfolding, microrheology

## Abstract

A detailed understanding of the mechanism of unfolding, aggregation, and associated rheological changes is developed in this study for β-Lactoglobulin at different pH values through concomitant measurements utilizing dynamic light scattering (DLS), optical microrheology, Raman spectroscopy, and differential scanning calorimetry (DSC). The diffusion interaction parameter *k*_D_ emerges as an accurate predictor of colloidal stability for this protein consistent with observed aggregation trends and rheology. Drastic aggregation and gelation were observed at pH 5.5. Under this condition, the protein’s secondary and tertiary structures changed simultaneously. At higher pH (7.0 and 8.5), oligomerizaton with no gel formation occurred. For these solutions, tertiary structure and secondary structure transitions were sequential. The low frequency Raman data, which is a good indicator of hydrogen bonding and structuring in water, has been shown to exhibit a strong correlation with the rheological evolution with temperature. This study has, for the first time, demonstrated that this low frequency Raman data, in conjunction with the DSC endotherm, can be been utilized to deconvolve protein unfolding and aggregation/gelation. These findings can have important implications for the development of protein-based biotherapeutics, where the formulation viscosity, aggregation, and stability strongly affects efficacy or in foods where protein structuring is critical for functional and sensory performance.

## 1. Introduction

The dynamic relationship between protein folding/unfolding and self-assembly (aggregation) has important implications in multiple areas of research and product development, which span from biotherapeutics and neurodegenerative diseases to additives in foods. For example, even though Alzheimer’s afflicts about 10% of the population over age 70 [1], the mechanism that triggers the misfolding of β-amyloid proteins and their subsequent aggregation into large plaques in brain cells is largely unknown. In the biotherapeutic world, where the development of stable protein formulations is constantly being sought, an understanding of the causes of protein aggregation would be advantageous to increase the drug’s bioavailability and thereby its efficacy [2]. Additionally, the consumer industry has a need to manipulate the structure and self-assembly of proteins in foods because of their impact on the overall rheological properties which give rise to desirable texture and flavor for the consumer.

The necessity for understanding protein folding and self-assembly has led to numerous studies on a wide range of proteins [3,4]. However developing a comprehensive understanding between colloidal and conformational dynamics in proteins has been challenging because the thermodynamic pathways that drive them, such as unfolding and aggregation, can occur simultaneously and, thereby, in a convoluted manner. Part of this challenge arises from the inability of analytical instruments to effectively tease apart and distinguish these convoluted unfolding and aggregation pathways.

To work toward successfully deconvolving these pathways, a combination of complementary techniques should be employed that can especially analyze several key properties of a single sample in tandem for more consistent results. Therefore, in this study a detailed concomitant dynamic light scattering (DLS), DLS microrheology, and Raman spectroscopy thermal aggregation study on a protein was carried out at a number of different pHs to explore the effects on both colloidal and conformational stability and associated rheological changes. Furthermore, in addition to Raman spectroscopy, differential scanning calorimetry (DSC) studies were carried out to explore the unfolding processes.

Colloidal stability plays a critical role in protein aggregation. The impact of weak protein–protein interactions on colloidal stability has been investigated for a number of protein systems [5]. In this regard, the second osmotic virial coefficient *B*_22_ has been explored as a potential predictor for colloidal stability and as a link to the solvent-mediated interaction of a pair of proteins. This parameter is averaged over the separation distance and relative orientations. When the interaction between the pair is primarily repulsive in nature (e.g., in conditions where the proteins are highly charged and in a low ionic strength environment), *B*_22_ exhibits a positive value while under conditions where the interactions are primarily attractive (e.g., near the isoelectric point), *B*_22_ exhibits a negative value. The diffusion interaction parameter (*k*_D_) has served as a surrogate for *B*_22_ because they exhibit a linear relationship with each other and the former can be directly obtained from DLS measurements with experimental ease and amenability to high-throughput measurements. The diffusion interaction parameter, *k*_D_, has been shown to be a good colloidal stability predictor for therapeutic proteins, such as monoclonal antibodies [6,7,8]. To the best of our knowledge, this understanding has, however, not been extended to food whey proteins, such as β-Lactoglobulin.

β-Lactoglobulin (βlg) is a milk protein with a molecular weight of 18.4 kDa and an isoelectric point around 5.13. It is known to exist as a dimer at neutral pH and dissociate into its constituent monomers below pH 3 [9]. Like many proteins, the self-association properties of βlg are strongly influenced by electrostatic interactions. The pH and ionic strength of the solution therefore play a critical role in the self-association behavior [10] which, for βlg, has been well investigated as a function of temperature [10,11,12,13,14] and ionic strength [15,16,17,18,19]. These studies have illustrated that a particular self-association route is dependent on both the pH and ionic strength, resulting in different onset temperatures for thermal aggregation, as well as different sizes and morphologies of the resulting aggregates. For example, the formation of rod-like aggregates, spherical aggregates, or worm-like aggregates have been reported based on the pH [20] and ionic strength [21] of the solution. Fibril formation has also been reported dependent upon formulation conditions [19,22,23,24,25,26,27,28,29]. A detailed comprehensive investigation on the effect of pH on the colloidal stability, as revealed through *k*_D_ via DLS, and its corresponding impact on conformational stability via DSC and Raman spectroscopy, has not been reported for this system [30,31]. Additionally the impact of the colloidal/conformational stability on the rheological response of the system has not been previously investigated systematically as a function of pH.

## 2. Results and Discussion

### 2.1. Evaluating the Colloidal Stability of βlg

DLS can quantify the propensity for aggregation and also the degree of interaction between individual proteins through the value of *k*_D_. The onset of aggregation and the extent of aggregation can also be further quantified by following changes in the hydrodynamic diameter (which in DLS is represented by the intensity weighted harmonic mean size, or *Z*-average) over time and under multiple conditions. In the current work, the colloidal stability of 4% *w*/*w* βlg was analyzed over a temperature range starting from 50 to 90 °C (incremented by 1 °C) at three pHs: (1) 20 mM sodium citrate and sodium phosphate (pH 5.5); (2) 20 mM sodium phosphate (pH 7.0); or (3) 20 mM Tris (pH 8.5). The effect pH has on colloidal stability over a temperature range was determined by DLS.

**Figure 1:**
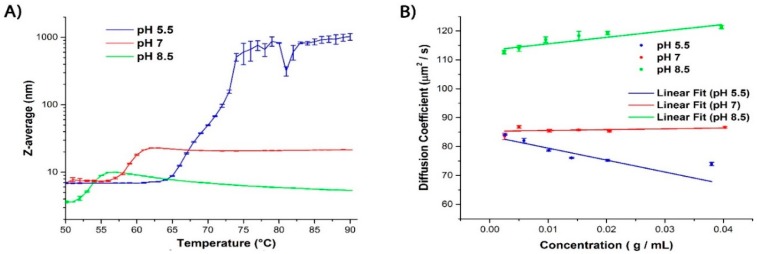
(**A**) Graph depicting changes in the *Z*-average diameter (*n* = 3) as a function of temperature and pH. At pH 5.5, the onset of aggregation, which is illustrated by a dramatic increase of the *Z*-average size, is observed at temperatures above ~65 °C. At temperatures above 74 °C, the protein solution exhibits non-ergodic behavior as it enters the gel state, making data acquisition by DLS unreliable. For pH 7.0 and 8.5 the aggregation effect is far less pronounced; and (**B**) Graph illustrating the change in the diffusion coefficient as a function of the three samples’ concentration. Each *k*_D_ value was determined from the slope of the linear fit to the data points. The *k*_D_ values become more positive with increasing pH.

After incubating each of the three samples for 30 min at each temperature, DLS measurements revealed that the *Z*-average begins to increase significantly after reaching a critical temperature (*T*_agg_) in all three cases. (Figure 1A and Table 1). It can be further seen that *T*_agg_ increases with decreasing pH and that the extent of the size increase is greater at the lower pH conditions, with pH 5.5, exhibiting a very steep increase in size after reaching the aggregation temperature. This indicates that the extent of self-association/aggregation increases with decreasing pH even though higher temperatures were necessary to initiate the process (Figure 1A and Table 1).

The ability to gain insights into the aggregation propensity of proteins and to monitor how that is impacted by formulation conditions is critical in order to optimize the formulation conditions for stability. As discussed in the introduction, *k*_D_ has been shown in therapeutic proteins [32] to be a good surrogate for *B*_22_ as a colloidal stability indicator and can be obtained from DLS measurements from the slope of the mutual diffusion coefficient *versus* protein concentration through use of the following relationship (Equation (1)):

(1) $$D=D_{0}\left[1+k_{D}c\right]$$

Figure 1B shows the change in the diffusion coefficient *versus* protein concentration for the three investigated pH values. The slopes of the corresponding least-squares fit yield the *k*_D_ values (Table 1) which become increasingly more negative with decreasing pH, indicating that the protein sample is becoming less colloidally stable. Since the isoelectric point of βlg is 5.13, the repulsive electrostatic interactions are greatly reduced when measured at pH 5.5, leading to stronger attractive interactions, and thus a higher propensity for aggregation. It has been shown that, at higher pH, the dominating repulsive interactions lead to more colloidal stability and base-induced denaturation causes dissociation of the dimer at room temperature [33]. This observation is consistent with the DLS data reported here (Figure 1A). Therefore, *k*_D_ is a good indicator of colloidal stability for these βlg solutions and that the protein exists in the monomeric state at pH 8.5 and as a dimer at pH 7.0 and pH 5.5.

**Table 1:** Relationship between pH and degree of aggregation.

pH	*T*_agg_ (°C) ^a^	*k*_D_ (L/mg) at 25 °C	*Z*-Average (nm)
5.5	65	−4.13	6.94
7.0	56	0.28	6.87
8.5	52	2.24	3.63

^a^ Temperature of the onset of aggregation.

Even though DLS can evaluate the colloidal stability of a protein solution, it alone does not provide a complete picture when used as a sole diagnostic of formulation stability. For example, while it is highly sensitive to the initial formation of high molecular weight oligomers and aggregates (physical changes), it is not able to untangle the additional structural (chemical) changes that either trigger or are a result of this aggregation.

### 2.2. Evaluating the Conformational Stability of βlg

#### 2.2.1. Differential Scanning Calorimetry (DSC)

DSC evaluates the thermodynamic stability of proteins during controlled heating by providing the enthalpy of unfolding and thermal transition midpoint (*T*_m_), where a higher *T*_m_ indicates higher stability. In addition to *T*_m_, DSC also derives other important thermodynamic parameters that can provide useful insights into the mechanism of unfolding, such as by comparing the calorimetric and van’t Hoff enthalpies [34] derived from each endotherm.

**Figure 2:**
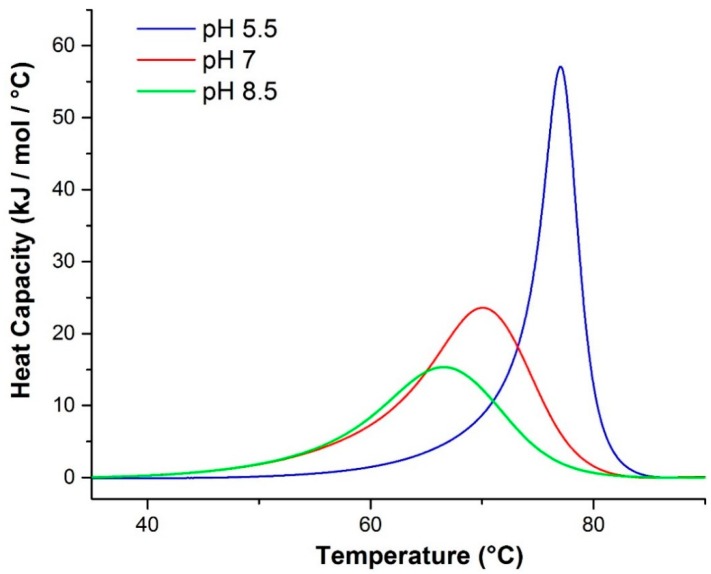
DSC endotherms of βlg as a function of pH. *T*_m_ increases with decreasing pH.

The three samples of βlg at pH 5.5, 7.0 and 8.5 were measured by DSC using a 1 °C/min scan rate. The resulting endotherms show a trend of enhanced stability with decreasing pH (Figure 2), which has been observed previously [35]. Using a Levenberg–Marquardt non-linear least-squares method with a single non-2-state model, the *T*_m_ values were extracted from each endotherm (Table 2).

The van’t Hoff (∆*H*_v_) and calorimetric (Δ*H*_cal_) enthalpies extracted from the fits to each endotherm are shown in Table 2. At pH 5.5, the ratio of van’t Hoff to calorimetric enthalpy is significantly larger than for the other two pH scans which are approximately unity in both cases. This suggests that the endotherm recorded at pH 5.5 is more likely the result of something more than a simple unfolding process. This is consistent with the DLS data (Figure 1) which had previously indicated significant aggregation and gelation at only this pH.

**Table 2:** Thermodynamic values obtained from Raman spectroscopy and DSC.

pH	*T*_m_ (°C) ^a^	Δ*H*_v_ (kJ/mol) ^a^	Δ*H*_cal_ (kJ/mol) ^a^	Δ*H*_v_^a^:Δ*H*_cal_^a^	*T*_m_ (°C) ^b^	*T*_m_ (°C) ^c^	Δ*H*_v_ (kJ/mol) ^d^	Δ*H*_v_ (kJ/mol) ^e^
5.5	76.7	711.3	298.9	2.380	70.5	72.1	520	757
7.0	69.2	281.8	316.6	0.8901	62.9	66.0	364	434
8.5	66.0	250.1	232.0	1.078	59.1	62.0	171	250

^a^ Thermodynamic values were extracted from the DSC endotherms (Figure 2) by performing a single non-2-state fit; ^b^ The temperature at which 50% of the original α-helical content is lost during heating as determined by Raman spectroscopy using a single sigmoid fit; ^c^ The temperature at which 50% of the original β-sheet content is gained during heating as determined by Raman spectroscopy using a single sigmoid fit; ^d^ van’t Hoff enthalpies for α-helix unfolding determined from the Raman data; ^e^ van’t Hoff enthalpies for β-sheet unfolding determined from the Raman data.

#### 2.2.2. Raman Spectroscopy

**Figure A1:**
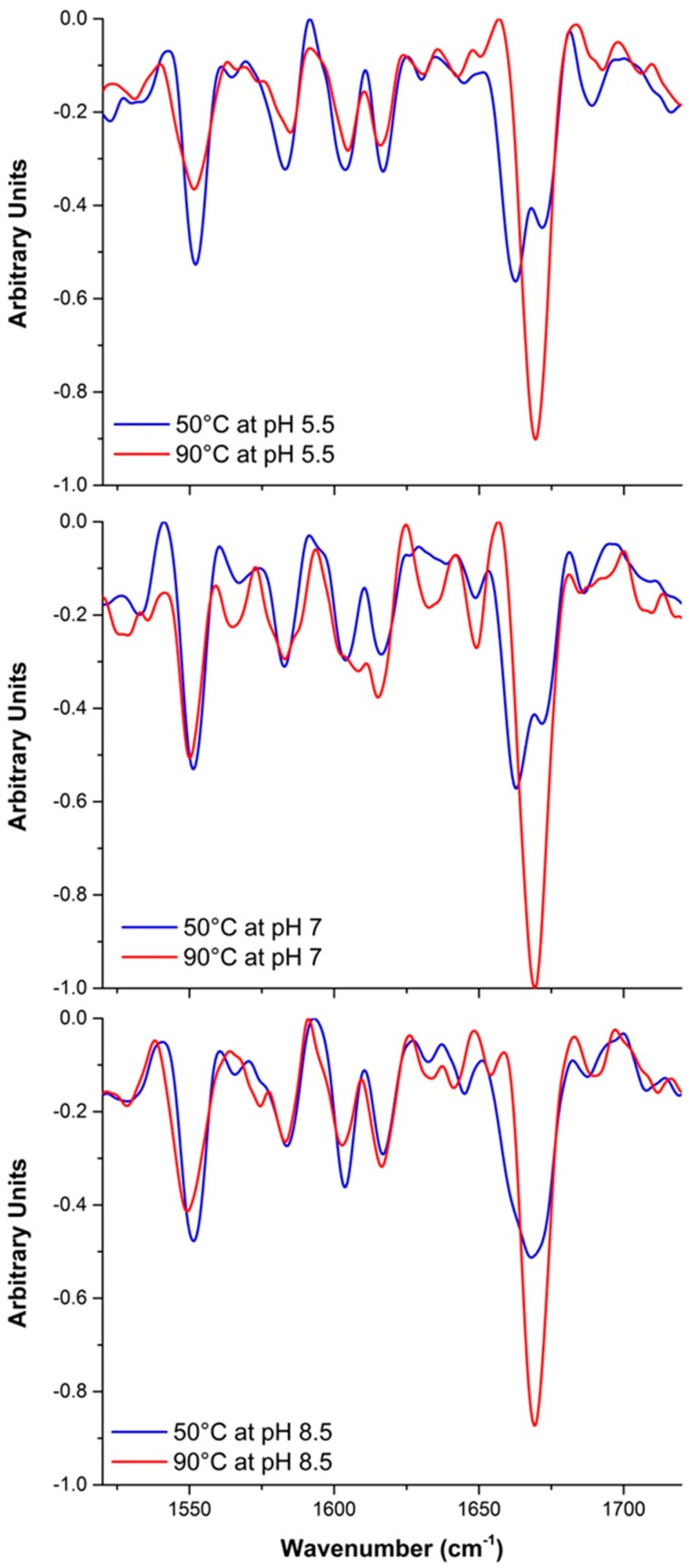
Typical Raman spectra from where data reported here is extracted.

In order to further understand the specific transitions that were observed in the DSC endotherms and DLS data, Raman spectroscopy was employed to probe the molecular level changes that underlie the thermal unfolding and aggregation of the protein (Figure A1).

**Figure 3:**
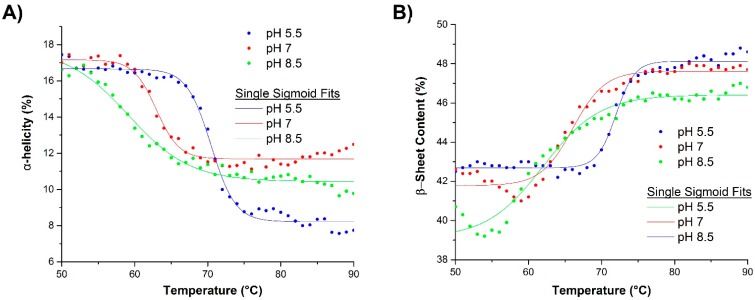
Change in the (**A**) α-helical and (**B**) β-sheet fraction as a function of temperature and pH. Content is observed at cooler temperatures.

During thermal ramping, the reorganization of the hydrogen bonding network within the protein results in changes in the protein’s secondary structure, such as with the α-helical, β-sheet, and random coil fractions. These values can be extracted directly from Raman spectra of the protein solutions [30,31,36,37]. Specifically, the secondary structure was determined as a function of pH during a thermal ramp (Figure 3), with all three protein samples exhibiting an unfolding and loss of α-helix as a function of temperature (Figure 3A). Similar to the results shown for the DSC data, the lower pH samples had higher unfolding temperatures. A similar (inverse) trend was also observed for the β-sheet fraction (Figure 3B) as a function of temperature. It should be further noted that while the α-helix fraction for all three pH conditions was initially similar, the pH 5.5 sample had a greater degree of total loss in α-helical content when compared to the other two pHs.

The van’t Hoff enthalpies for the loss of α-helix and gain in β-sheet fraction was determined at each pH using a single Sigmoid fit (Table 2). Both sets of values describe the intramolecular energetics associated with the unfolding event. Because of the large van’t Hoff enthalpy of the β-sheet fraction at pH 5.5 and the nature of that enthalpy to decrease with increasing pH, it can then be noted that the anomalous enthalpy at pH 5.5 results from the convolution of intermolecular β-sheet from aggregation and the formation of intramolecular β-sheet from unfolding.

**Figure 4:**
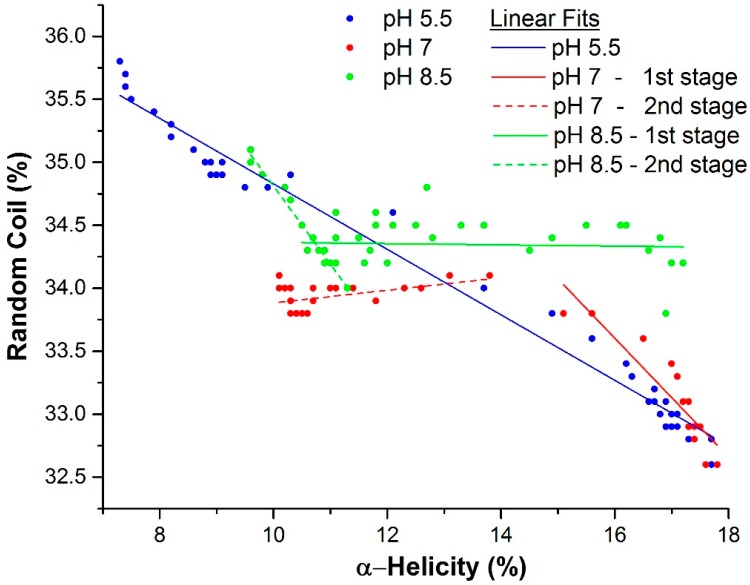
Random coil and α-helix structure of βlg plotted for all pH conditions and all temperatures. Linear fits to all or subsets of the data points support a model consistent with different mechanisms of unfolding for all three pH conditions.

Upon further analysis of the data acquired by Raman spectroscopy (Figure 4), different pathways in terms of changes in the secondary structure can be observed. pH 5.5 (blue points) shows a single cooperative interchange between fraction α-helix and random coil while at pH 7.0 (red points) and 8.5 (green points) this relationship is broken. Specifically, throughout the temperature ramp at pH 5.5, the loss in α-helical content is directly correlated with a cooperative increase in the random coil and β-sheet fractions. At pH 8.5, the change is initially mostly connected with increasing β-sheet fraction and more involvement of conversion to random coil at higher temperatures. At pH 7.0, a simultaneous transition to developing β-sheet and random coil from α-helix is initially observed which then collapses to mostly β-sheet development.

**Figure 5:**
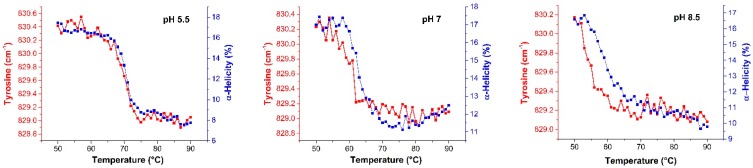
Raman data showing the frequency of the tyrosine (Tyr) peak (red) and α-helix fraction (blue) as a function of temperature for all three pH conditions. The position tyrosine peak is an indicator of a change in the tertiary structure of the protein and complements the Raman data derived for changes in the secondary structure.

To further correlate changes in the tertiary with those observed for the secondary structure, the frequency of the tyrosine (Tyr) marker at ~830 cm^−1^ was also simultaneously monitored (Figure 5). Raman frequencies of aromatic amino acid are sensitive to changes in their solvent environment (hydrophobic *versus* hydrophilic) and, therefore, monitor changes in the tertiary structure [31,37,38]. For pH 5.5, the frequency shift took place almost simultaneously with the change in the α-helical content, indicating that the changes in the protein’s tertiary and secondary structure occur simultaneously. At pH 8.5, the Tyr marker transitioned much earlier than the change in the α-helical content over the same temperature range, indicating that a transition in the tertiary structure occurs before the change in the secondary structure. At pH 7.0, the relative change between the secondary and tertiary structure was intermediate to that seen for pH 5.5 and 8.5. If these insights from Raman spectroscopy are combined with the DLS observations (Figure 1A), which shows a monomeric state at pH 8.5 and a multimeric state at pH 5.5 and 7.0, the data suggests that at a higher pH (furthest away from the p*I*), βlg undergoes a tertiary structural change and the formation of a dimer, or higher order oligomer, prior to the onset of significant aggregation and further unfolding.

### 2.3. Mechanism of Unfolding and Aggregation

#### 2.3.1. Correlation between Protein Structural Changes and Microstructure/Rheology

**Figure 6:**
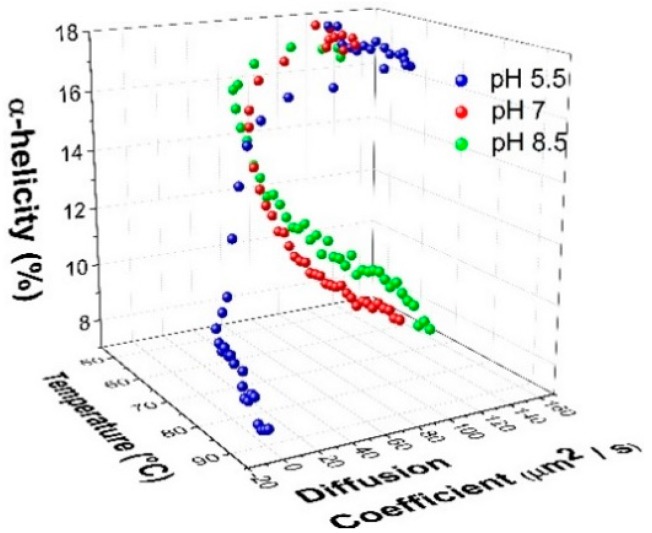
Correlation of the change in the α-helical content from Raman spectroscopy with the diffusion coefficient from DLS as a function of pH and temperature.

By plotting the changes in the diffusion coefficient as a function of temperature and change in the α-helix fraction (Figure 6), it can be shown that pH 5.5 exhibits a rapid decrease in both the diffusion coefficient and α-helical content with increasing temperature. The diffusion coefficient approaches zero, indicating an arrested state or gelation taking place.

**Figure 7:**
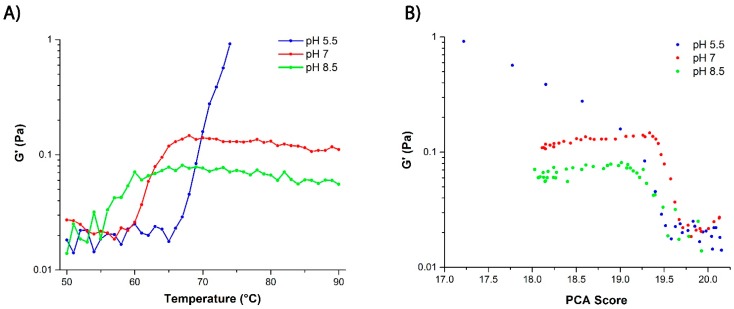
(**A**) Graph depicting the change of the elastic modulus (*G*′) measured by DLS microrheology as a function of pH and temperature. The pH 5.5 sample showed the largest elastic modulus. Beyond 74 °C, the protein solution at pH 5.5 exhibits non-ergodic behavior as it enters the gel state, making data acquisition by DLS unreliable; (**B**) Elastic modulus plotted *versus* changes in the principal component analysis (PCA) score derived from the low frequency (intermolecular) Raman spectral interval (200–400 cm^−1^).

In order to further investigate this gelation effect, DLS-microrheology was carried out. In general, the elastic modulus (*G*′) acquired by optical microrheology is a strong indicator of microstructural evolution and network formation in self-assembling systems, such as agarose [39] and surfactant-based wormlike micelles [40]. Here, the evolution toward gelation is illustrated by the rapid increase in *G*′ as a function of temperature, especially at pH 5.5 (Figure 7A). The gelation can be attributed to aggregation of the protein at the higher temperatures at this pH. This observation is further validated when the elastic modulus is analyzed against the low-frequency Raman data that measures the enhanced hydrogen bonding in water and the degree of water confinement [39,40]. This portion of the Raman spectrum is seen to correlate well with the *G*′ evolution (Figure 7B). It should be noted that the increase in *G*′ for the other two pHs is minimal, indicating there’s no progression to a gel or an arrested state.

#### 2.3.2. Raman Spectroscopy as a Means for Studying the Relative Interactions of Protein Unfolding and Aggregation

**Figure 8:**
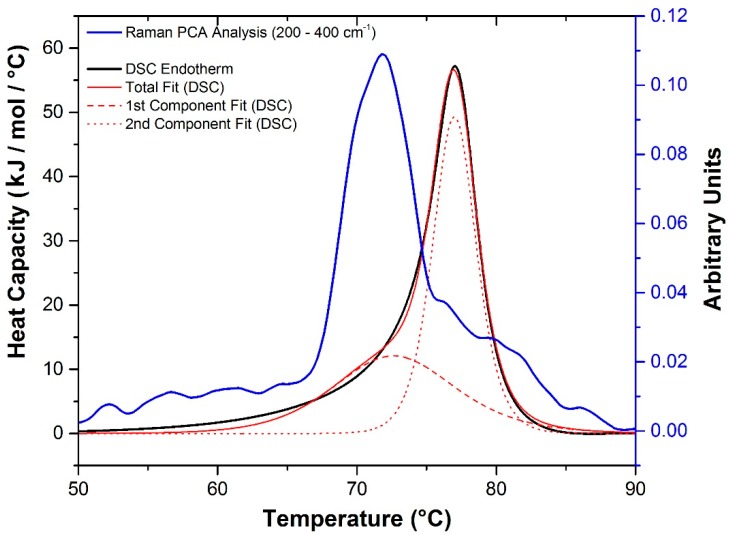
Derivative of the change in the Raman PCA score (200–400 cm^−1^) *versus* temperature (blue line), for the sample at pH 5.5, indicative of changes in intermolecular interactions and the DSC endotherm of the sample at pH 5.5 (black line). Double non-2-state fit of the DSC data (red solid, dashed and dotted lines).

Previous work has shown a link between the low-frequency Raman data (<400 cm^−1^) and evolution of the elastic modulus in a number of model systems [39,40]. These changes are assigned to modifications of hydrogen bonding in water and the degree of water confinement in gels and other colloidal systems. As such, it provides a good probe of the intermolecular interactions and aggregation in proteins. Correlating DSC data with information derived from this Raman spectral interval has the potential to help deconvolve multiple thermodynamic events that can occur during a DSC experiment. Using a Levenberg–Marquardt non-linear least-squares method with a double non-2-state model, two *T*_m_ values were extracted from the DSC endotherm at pH 5.5 (Figure 8). These data are suggestive of an intermediate step prior to the main unfolding event. Plotting this data alongside the score derived from a principal component analysis (PCA) of the Raman spectral interval between 200–400 cm^−1^ *versus* temperature reveals the likelihood that this is the result of an additional intermolecular process (aggregation) which initially precedes and eventually thermally overlaps with molecular unfolding.

## 3. Experimental Section

A Zetasizer Helix (patent pending) from Malvern Instruments (Malvern, UK) has been used to obtain DLS (nanoscale particle size) and Raman (structural information) data sequentially on a single sample. The Helix system uses a proprietary non-invasive backscatter (NIBS) detector (Malvern Instruments, Malvern, UK) with dynamic (DLS), static (SLS) and electrophoretic (ELS) light scattering to measure the hydrodynamic radius of particles from 0.15 nm to 5 µm. Raman spectra are collected using 785 nm excitation (~280 mW) from 150 to 1925 cm^−1^ at 4 cm^−1^ resolution.

β-Lactoglobulin (βlg) (obtained in 90% purity from Sigma-Aldrich Corp. (St. Louis, MO, USA)) was dissolved at 40 mg/mL in a solution of one of the following three pHs: (1) 20 mM sodium phosphate buffer (pH 7.0); (2) 20 mM sum of sodium phosphate and sodium citrate buffer pH 5.5; and (3) 20 mM Trizma base buffer (pH 8.5). After preparation, samples were filtered through 200 nm syringe filters and final concentration of βlg was confirmed using UV spectroscopy (Thermo Fisher Scientific, Waltham, MA, USA).

DLS, microrheology measurements and Raman spectroscopy were performed on the Helix instrument (Malvern Instruments, Malvern, UK) for the three aforementioned samples as well as their associated buffers for reference. Each sample was subjected to a temperature range from 50 to 90 °C at 1 °C intervals with a 30 min equilibration window at each temperature. After equilibration, data from each sample was collected over 15 min in triplicate for DLS, over 15 min for microrheology, and over a 30 s exposure time with 10 scans for Raman spectroscopy at each temperature. Sample aliquots (~20 µL) for Raman and DLS work were placed into a proprietary titanium cuvette with 120 µm thick quartz windows, and positioned in a temperature-stabilized sample holder. For microrheology experiments, 500 µm tracer particles that contain a silica core, and is surface-functionalized with polyethylene glycol polymers (Micromod, GmbH, Rostock, Germany), was added to the protein samples to final concentration of 0.15% *w*/*w* before each measurement. The percentage of secondary structure components were deconvolved from a Raman protein library of 18 model proteins with a PLS model. The accuracy of the library has been validated and confirmed within 5% deviation from commonly applied CD library or corresponding secondary structure percentage from Worldwide Protein Data Bank for the corresponding protein [41].

Differential scanning calorimetry (DSC) was performed using a MicroCal VP-Capillary DSC (Malvern Instruments, Northampton, MA, USA). Thermograms for each protein and buffer sample (350 μL at 40 mg/mL) were obtained from 25 to 100 °C using a scan rate of 60 °C·h^−1^. Thermograms of the buffer alone were subtracted from each protein prior to analysis using Origin 7.0 (OriginLab, Northampton, MA, USA) graphing software equipped with the MicroCal VP-Capillary DSC (Malvern Instruments, Malvern, UK) analysis software add-on. Fitting was done using a Levenberg–Marquadt non-linear least-squares method that specifically involved a non-2-state model.

## 4. Conclusions

This study has illustrated the strong effect of pH on both colloidal and conformational stability and associated rheological changes for βlg. At pH 5.5, the βlg undergoes a rapid decrease in the diffusion coefficient and more profound secondary structural changes with increasing temperature. Corresponding increases in the elastic modulus (*G*′) indicates that this system is undergoing gelation as a result of protein aggregation. At higher pH, equivalent changes in the diffusion coefficient and secondary structure are not seen. Significant changes in the elastic modulus *G*′ are also not observed. The melting transition for the pH 5.5 solution occurs at higher temperatures as seen in the DSC measurements and we propose that this is due to a convolution of unfolding, aggregation and gelation. An attempt to deconvolve these effects has been undertaken through resolving the DSC melting transition into two events and comparing it with the low-frequency Raman data, which we assign to the development of an intermolecular hydrogen bonding network. These results further indicate that, at pH 5.5, βlg undergoes further structural changes after aggregation and gelation. Although this study provides a preliminary view of the differences in structural changes and their associated effect on aggregation and rheological changes for a gelling system *versus* a non-gelling system, a broader comprehensive understanding of the effect of pH requires an extension to both higher and lower pH values than investigated in this study. This is currently ongoing and will be the subject of a future publication. Overall, this study has shown that the combination of DLS, Raman spectroscopy, microrheology, and DSC allows a more comprehensive understanding of the unfolding mechanisms and self-assembly mechanisms in protein systems like βlg.
